# Supercritical carbon dioxide extraction of Usnea longissima (L.) Ach.: Optimization by Box-Behnken design (BBD)

**DOI:** 10.3906/kim-2102-66

**Published:** 2021-08-27

**Authors:** Ceren ATİLA DİNÇER, Ceren GÖKALP, Bengü GETİREN, Atila YILDIZ, Nuray YILDIZ

**Affiliations:** 1 Department of Chemical Engineering, Faculty of Engineering, Ankara University, Tandoğan, Ankara Turkey; 2 Department of Biology, Ankara University, Ankara Turkey

**Keywords:** *Usnea longissima* (L.) Ach., usnic acid (UA), supercritical extraction, 9response surface methodology (RSM)

## Abstract

Usnic acid (UA) was extracted from *Usnea longissima* (L.) Ach. in supercritical carbon dioxide (SC-CO_2_) medium. The selected process parameters were extraction temperature (35–45 °C), amount of co-solvent (0%–5%) and extraction time (5–9 h). These parameters were applied to Box-Behnken design (BBD) belonging to response surface methodology (RSM) to determine optimum process parameters for the highest amount of UA in the extract. g UA/100g lichen, extraction yield % and UA content values were calculated in the range of 0.045–0.317, 2.77–5.4 and 71%–82% in different experimental conditions, respectively. The optimum conditions were predicted as 42 °C, 4.3% (ethanol) and 7.48 h. It was determined that the predicted and experimental values of g UA/100g lichen were compatible, and the suggested model was valid.

## 1. Introduction

Usnic acid (UA) [2,6-diacetyl-7,9-dihydroxy-8,9b-dimethyl-1,3(2H,9bH)-dibenzo-furandione] is a derivative of low molecular weight dibenzofuran. This yellowish material exists among the secondary lichen metabolites. Lichens are symbiotic organisms, which comprise algae, fungus and/or cyanobacteria. Usnic acid found in various lichen structures such as *Alectoria, Cladonia, Evernia* , *Lecanora, Ramalina, Usnea and Xanthoparmelia* has antimicrobial properties and plays role as an ingredient in products like cream, deodorant, toothpaste, mouthwash and sunscreen. Besides, it has antiviral, analgesic, antiproliferative, anti-inflammatory, cytotoxic and antitumor properties [1–4].

The conventional extraction processes of plants and plants-like structures have some drawbacks due to high temperature, toxic solvent and extra separation treatments. To overcome these difficulties, nonconventional extraction techniques such as supercritical fluid extraction (SFE), ultrasound-assisted extraction, microwave-assisted extraction, pressurized liquid extraction and pulsed electric fields extraction methods are used. If these methods are compared in terms of energy consumption, SFE process requires less energy than other techniques. So, SFE is more economically suitable for research and industrial applications. While the other methods applied in extraction fractionation and separation processes have some disadvantages such as high energy costs, low selectivity and large quantities of solvent waste, supercritical fluid extraction has no these limitations. Besides that, applying high temperature in microwave-assisted extraction and pressurized liquid extraction methods are not suitable for thermolabile compounds. Since thermolabile compounds are not damaged at critical temperature and pressure, using the supercritical extraction method is more advantageous than other methods [5–7]. Carbon dioxide (CO_2_) is the most preferred solvent for SFE process. Supercritical carbon dioxide (SC-CO_2_) with mild critical values (31.1 °C and 7.28 MPa) and appropriate properties such as non-flammability, non-toxicity, high selectivity and cheapness is very suitable for food, pharmaceutical and environmental areas [8–10]. The studies about SC-CO_2 _extraction process with or without response surface methodology (RSM) were placed in the literature [11–15].

RSM is one of the most attractive experimental design method used by researchers in a number of chemical processes. The Taguchi method, central composite design (CCD) and Box–Behnken design (BBD) are the most commonly used experimental designs for optimization of supercritical fluid extraction in the literature. It is found that the Box-Behnken design proved to be much more favorable and efficient than the other response surface designs due to its effectiveness and use of three instead of five levels for each factor [16–18].

In the literature, there are some studies in which usnic acid to be used in different applications is obtained by traditional and supercritical extraction methods from different lichen species. Cansaran-Duman et al. [19] obtained usnic acid from several species lichens collected from different regions in Turkey. The extraction from lichens such as *Evernia illarica* , *Usnea barbata and Usnea longissima* was carried out with acetone. Usnic acid contents was found between 0.14–6.49 dry weight percentages according to lichen species. *Usnea barbata* lichens were extracted with SC-CO_2 _at different pressure (30–50 MPa) and temperature (25–40 °C) conditions by Ivanovic et al. [20]. SC-CO_2 _extraction process was applied after different pre-treatment methods, including roller-, ultracentrifugal- and cutting mill. The highest extraction yield and usnic acid content in the extract were found as 2.08% and 632 g/kg, respectively. Fanovich et al. [21] extracted *Usnea lethariiformis* using SC-CO_2 _at 30 MPa and 40°C, and the usnic acid content in the extract was found as 50% (w/w). The lichen extract was used for impregnation of polycaprolactone scaffolds to determine antibacterial activity. Koçer et al. [22] obtained usnic acid from *Usnea longissima* lichen (1500 g) extract by refluxing dichloromethane. They synthesized hydroxyphenylimino ligands and their complexes with usnic acid and investigated antimicrobial and antimutagenic activities of synthesized materials. Zugic et al. [23] studied *Usnea barbata* (Old Man’s Beard) extraction with supercritical fluid (E1) and conventional (ether fraction E2, ethanol fraction E3 and macerate E4) methods. Usnic acid content (w/w%) was determined as 81.41, 67.09, 2.43 and 1.39 with E1, E2, E3 and E4 methods, respectively. The lichen extract was used to examine anticancer and antioxidant activities.

In this work, *Usnea longissima* (L.) Ach. was extracted by using supercritical carbon dioxide (SC-CO_2_) extraction, and the Response Surface Methodology (RSM) Box-Behnken Design (BBD) was realized to define the optimum process parameters of usnic acid extraction. To the best of our knowledge, this is the first study related to SC-CO_2 _extraction from *U. longissima* and optimization of this process by RSM.

## 2. Materials and methods

### 2.1. Materials

*Usnea longissima* (L.) Ach. was collected from Fırtınaderesi/Zilkale-Rize (Turkey). CO_2_ of 99.9% was supplied from Linde Group (Ankara, Turkey). High purity ethanol and commercial usnic acid were purchased from Sigma Aldrich. 

### 2.2. Usnea longissima (L.) Ach. extraction 

**Figure 1:**
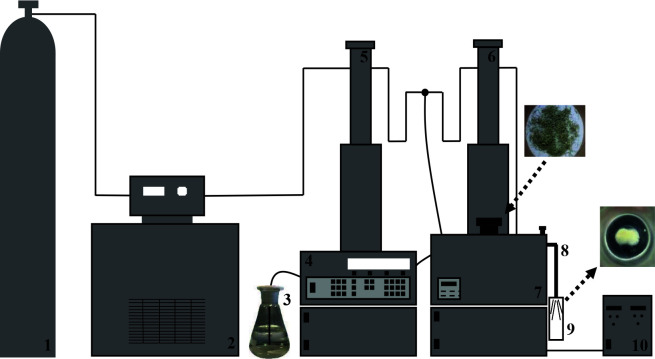
ISCO-Sitec modified SFX 220 supercritical fluid extraction system (1) CO_2_ storage, (2) circulator, (3) co-solvent storage, (4) controller, (5) CO_2_ pump, (6) co-solvent pump, (7) extraction unit, (8) capillary nozzle, (9) expansion vessel, (10) restrictor temperature controller.

The *U. longissima* extract was obtained by ISCO-Sitec modified SFX 220 supercritical fluid extraction system (Figure 1). Two high pressure syringe pumps (Model 100 DX, ISCO Inc., NE), extraction unit (SFX 220, ISCO Inc., NE) and controller unit (D-Series Pump Controller, ISCO Inc., NE) were essential parts of the supercritical system. One of the two syringe pumps was used for CO_2_ and the other for ethanol as co-solvent. 

In the experiments, 220 mg of *U. longissima* lichen was filled in 2.5 mL stainless steel extraction cell, and the loaded cell was placed in the extraction unit. The extraction temperature and pressure were set to the desired values. Extraction pressure (300 bar) and supercritical carbon dioxide flow rate (5.5 mL/min) were kept constant during the process. The system has been run as a batch process at certain periods to allow *U. longissima* extraction by supercritical carbon dioxide with or without co-solvent. Supercritical solution was expanded rapidly through a capillary nozzle with L = 3–4 mm, D = 50 μm, and the lichen extract was obtained in solid form (Figure 1).

### 2.3. Determination of usnic acid (UA)

The amount of UA in the supercritical extract was determined by HPLC analysis. The calibration solutions were prepared in acetone (0.5–50 mg/ L) with commercial usnic acid, and the extracts from *U. longissima* were analyzed by dissolving in acetone. Shim-Pack CLC-ODS column (4.6 × 250 mm; 5 µm) was used to detect UA and the system was operated with mobile phase PBS / methanol (30:70%), flow rate 0.8 mL/min, column temperature 30 °C, injection volume 20 µL and UV detector wavelength at 245 nm [24]. Each analysis was performed in triplicate.

Fourier-transformed infrared spectroscopy (FTIR, 8400 S Shimadzu) was applied using the KBr pellet method.

### 2.4. Experimental design

The response surface methodology (RSM) Box-Behnken Design (BBD) was applied (Design Expert software version 10.0.3.0, Stat-Ease) to determine the optimum process parameters of usnic acid extraction. RSM, which is used for modelling and analysis, includes a group of experimental techniques to find the relationship between controlled experimental independent variables and measured responses (dependent variables). This method allows several experiments to reach the most information at the certain points. In this study, extraction temperature (35–45 °C), amount of co-solvent (0%–5%), extraction time (5–9 h) were selected as independent variables based on the pre-experiments, and the response variable was the amount of UA (g) for 100 g lichen (g UA/100g lichen). The relation between the actual and coded values of the independent variables was expressed by following equation (1):

(1)xi=Xi-X0ΔXi

where xi symbolizes the coded value, Xi represents the actual value, X_0_ is the actual value at the center point and ΔX_i_ is the step change of the actual value. The actual and coded values of the independent variables are given in Table 1. 

**Table 1:** Coded and actual independent variables.

Symbol	Variables	Coded and actual levels		
		–1	0	1
x1	Extraction temperature (°C)	35	40	45
x2	Amount of co-solvent (%)	0	2.5	5
x3	Extraction time (h)	5	7	9

RSM Box-Behnken design (BBD) suggested 17 experiment for three independent variables. The response variables were correlated into the second-order polynomial given in equation (2): 

(2)Y=β0+∑i=13βixi+∑i=13βiixi2+∑∑i<j3βijxixj

where *Y* is indicates the response, β *_0_* is a constant, β *_i_* , ** β *_ii_* and β *_ij_* are the linear, quadratic and interactive coefficients, *x* *_i_* and *x* *_j_* represent variables. Analysis of variance (ANOVA) was applied to analyze the result based on the significance level of 0.05 [25–27].

## 3. Results and discussion 

**Figure 2:**
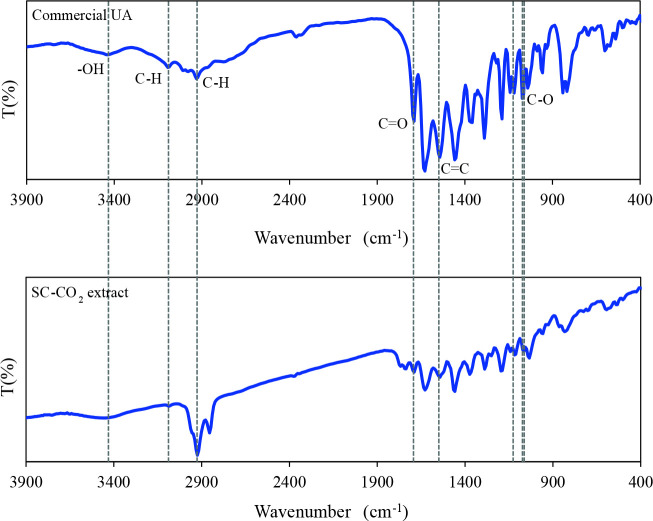
FTIR spectra of commercial UA and supercritical extract (40  C, 2.5%, 7h).

FTIR spectra of commercial UA and supercritical lichen extract (40 °C, 2.5%, 7h) are given in Figure 2. When the FTIR spectrum of commercial usnic acid were examined, -OH: 3441 cm^–1^, C = O: 1689 cm^–1^, C = C: 1543 cm^–1^, CO: 1072, 1118, 1141 cm^–1^, CH (aromatic): 3093 cm^–1^ and CH (aliphatic): 2931 cm^–1^ characteristic peaks [28–29] were observed. In the FTIR spectrum of the SC-CO_2_ extract (40 °C, 2.5%, 7h) (-OH: 3448 cm^–1^, C = O: 1689 cm^–1^, C = C: 1543 cm^–1^, CO: 1064, 1118, 1141 cm^–1^, CH (aromatic): 3086 cm^–1^ and CH (aliphatic): 2924 cm^–1^) similar peaks to the commercial structure were obtained. It is observed that the structure of the supercritical extract and commercial UA were compatible with each other.

**Figure 3:**
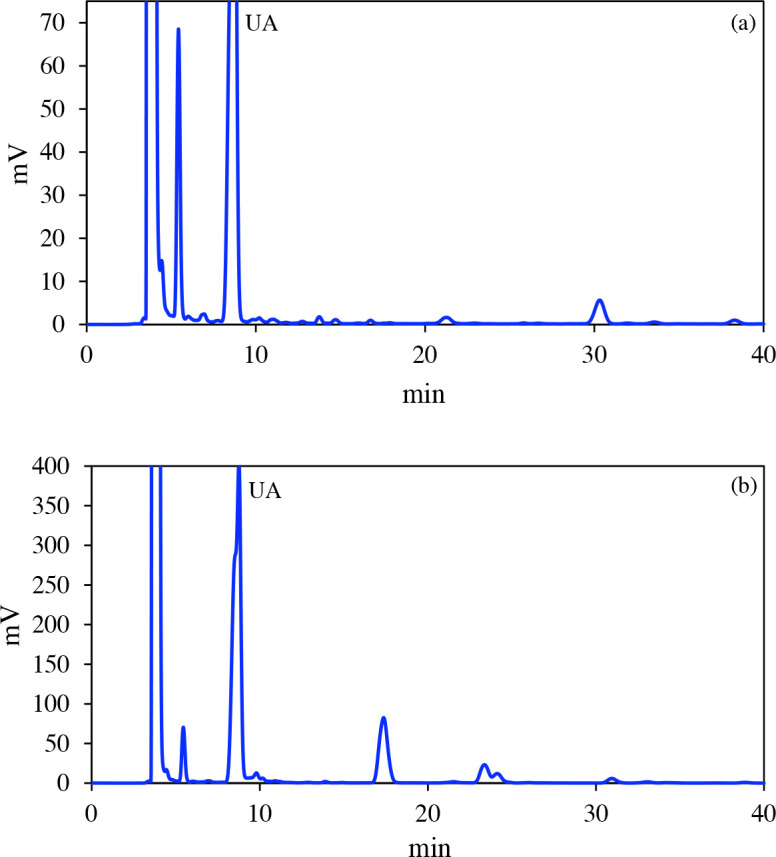
HPLC chromatograms of the (a) calibration solution (50 mg / L) (b) center point experiment (40  C, 2.5%, 7h).

The extraction yield (%), UA content (%) and g UA/100g lichen values were calculated in the experiments carried out under the conditions determined by the RSM (Table 2). The extraction efficiency was calculated from the differences between initial and final amount of lichen during the extraction process, and the UA content (%) was found from the peak ratio obtained by HPLC chromatograms. HPLC chromatograms of the samples of the calibration solution (50 mg / L) and center point experiment (40 °C, 2.5%, 7h) are given in Figure 3. 

**Table 2:** Experimental program using Box-Behnken design, response and calculated extraction values.

		Factor 1	Factor 2	Factor 3	Response			
Std	Run	x_1_:Extr. temp. (°C)	x_2_:Amount ofco-solvent (%)	x_3_:Extr. time (h)	g UA/ 100 g lichen	Amount of UA (mg)	Extraction yield (%)	UA content (%)
10	1	40	5	5	0.290	0.639	4.54	71.87
14	2	40	2.5	7	0.302	0.665	4.13	73.64
9	3	40	0	5	0.271	0.596	3.77	77.24
8	4	45	2.5	9	0.171	0.377	4.91	73.91
17	5	40	2.5	7	0.317	0.697	4.31	71.34
12	6	40	5	9	0.295	0.650	3.22	77.12
13	7	40	2.5	7	0.266	0.586	4.22	71.41
7	8	35	2.5	9	0.045	0.098	4.90	76.30
5	9	35	2.5	5	0.077	0.170	3.54	75.50
4	10	45	5	7	0.297	0.654	5.40	82.57
11	11	40	0	9	0.051	0.113	3.68	77.02
16	12	40	2.5	7	0.286	0.629	3.45	74.84
6	13	45	2.5	5	0.184	0.404	4.81	74.95
15	14	40	2.5	7	0.302	0.665	4.13	73.64
2	15	45	0	7	0.223	0.490	4.72	82.37
1	16	35	0	7	0.071	0.156	2.77	73.71
3	17	35	5	7	0.150	0.330	3.59	80.42

Extraction yield%, UA content and g UA/ 100g lichen values were calculated in the range of 2.77–5.4, 71%–82%, 0.045–0.317, respectively. These values were compared with limited studies of the lichen extraction by SC-CO_2_ in the literature. Zizovic et al. [30] found the extraction yield as 0.38% and 0.60%, usnic acid content as 59.48% and 36.49%, and g UA/100g lichen as 0.219 and 0.226, respectively. Brovko et al. [31] obtained 91% UA content by evaluating the peaks in the HPLC chromatogram for the first 10 min. In this study, the duration of the analysis was extended (40 min), and the peaks of the possible components except the UA were considered. The UA content was determined as 71%–82%. No purification was required due to the high percentage of UA content in the extract. 

The results of the variance analysis (ANOVA) of the quadratic model used for the optimization of the UA extraction parameters were given in Table 3. In addition, the polynomial equation was presented below:

**Table 3:** The results of the ANOVA of the quadratic model.

Source	Sum of squares	df	Mean square	F value	p-value Prob > F
Model	0.15	9	0.017	16.32	0.0007
x_1_	0.035	1	0.035	34.53	0.0006
x_2_	0.022	1	0.022	21.11	0.0025
x_3_	8.450E-003	1	8.450E-003	8.25	0.0239
x_1_x_2_	6.250E-006	1	6.250E-006	6.100E-003	0.9399
x_1_x_3_	9.025E-005	1	9.025E-005	0.088	0.7752
x_2_x_3_	0.013	1	0.013	12.35	0.0098
x_1_^2^	0.049	1	0.049	48.31	0.0002
x_2_^2^	3.603E-006	1	3.603E-006	3.516E-003	0.9544
x_3_^2^	0.019	1	0.019	18.40	0.0036
Residual	7.173E-003	7	1.025E-003		
Lack of fit	5.670E-003	3	1.890E-003	5.03	0.0764
Pure error	1.503E-003	4	3.758E-004		
Cor total	0.16	16			
R^2^	0.9545				
R^2^_Adj_	0.8960				

g UA/ 100g lichen = 0.29 + 0.067*x_1 _+ 0.052*x_2 _– 0.033*x_3 _– 1.250E-003*x_1_x_2_+ 4.750E-003 x_1_x_3 _+ 0.056*x_2_x_3_– 0.11*x_1_^2^– 9.250E-004*x_2_^2 ^+ 0.067*x_3_^2^ (3)

Probe > F values less than 0.05 indicated that the model parameters were significant. The F test and Probe > F values of the model were found as F = 16.32 and Probe > F = 0.0007, which shows that the model was significant. According to ANOVA results, x_1_, x_2_, x_3_,x_2_x_3_, x_1_^2^ and x_3_^2^model parameters had an important effect on the g UA/100g lichen. The regression coefficient (R^2^) indicated how the relation between the independent variables was expressed by suggested model. R^2^ and R^2^_adj_ (adjusted regression coefficient) values were determined as 0.9545 and 0.8960, respectively. The value of the lack of fit was found as not significant (p = 0.0764). This indicated that the model fitted the data well [32]. The BBD model was acceptable with considering all these values. When the equation coefficients were examined, it was seen that the increase of extraction temperature (x_1_) and amount of co-solvent (x_2_) causes the g UA/ 100g lichen to increase, while the increase of extraction time (x_3_) had a negative effect on the g UA/ 100g lichen. The increasing of the temperature at constant pressure leads to decrease of the density of the supercritical fluid. On the other hand, the increase of the vapor pressure of the solute causes to increase the solubility of the solute. The addition of co-solvent (ethanol) to apolar compound carbon dioxide medium enhances the solubility of the polar compounds in the supercritical medium. The long extraction time can cause degradation of compound or conversion to other compounds [33–35].

**Figure 4:**
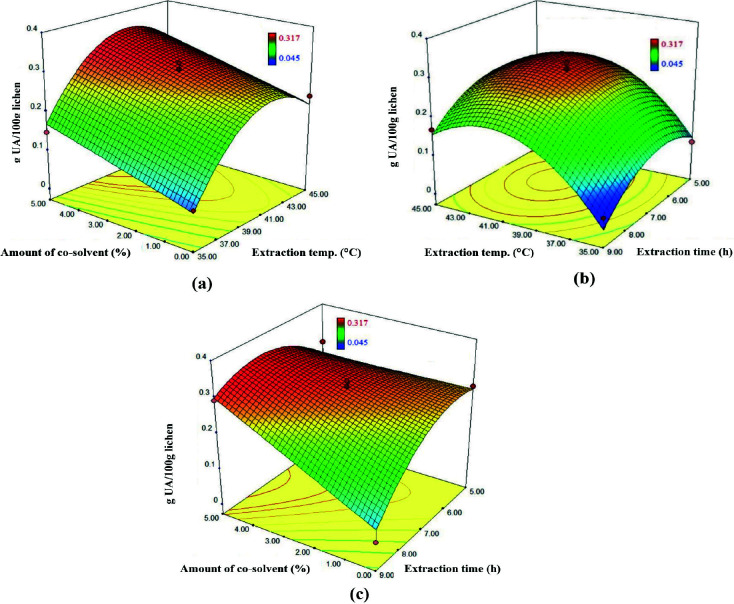
3D response surface plots of the g UA/100g lichen affected by (a) amount of co-solvent and extraction temperature (b) extraction temperature and extraction time (c) amount of co-solvent and extraction time.

In Figure 4, the binary effects of the independent variables on the response were given graphically. Amount of co-solvent/ extraction temperature binary interaction was determined as not significant (p = 0.9399) in Table 3. However, the 3D graph (Figure 4a) shows that this binary interaction passes through an apparent maximum ridge. While the extraction temperature makes the Y variable between 41–43°C maximum, the amount of co-solvent does not cause a significant change; however, as its amount increases, it makes an increasing contribution to the Y variable. Extraction temperature/ extraction time binary interaction was determined as not significant (p = 0.7752). On the other hand, the 3D graph (Figure 4b) indicates that this binary interaction has gone through a significant circular maximization. The extraction temperature and the extraction time make the Y variable maximum between 41–43°C and 7–8 h, respectively. Amount of co-solvent/ extraction time binary interaction was determined as significant (p = 0.0098) in Table 3. The 3D graph (Figure 4c) shows that this binary interaction passes through an apparent maximum ridge. The values at which the extraction time makes the Y variable maximum change within 7–8 h. While the Y variable is affected negatively in the range of 8–9 h of extraction time, Y variable increases by increasing of amount of co-solvent. According to the statistical results, it was determined that the extraction temperature and amount of co-solvent are more positive effective parameters than extraction time on the Y variable. 

**Figure 5:**
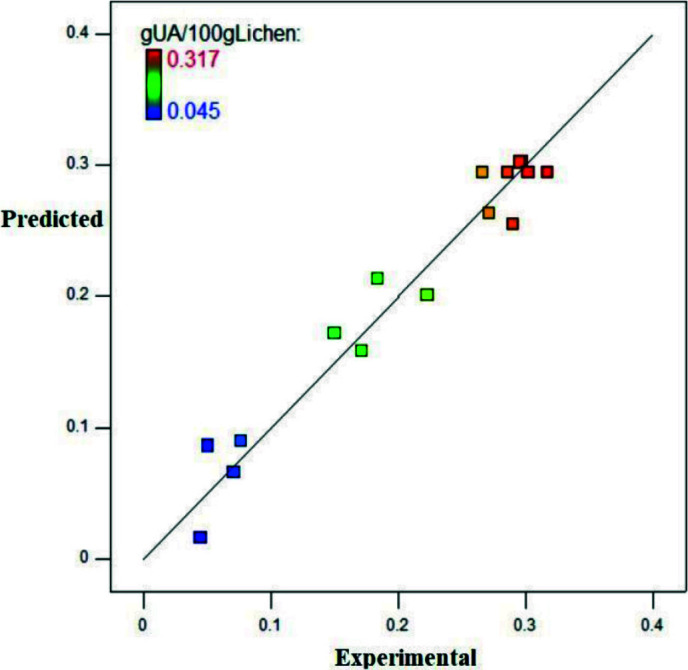
Comparison of experimental and predicted values of the g UA/100g lichen.

The optimum conditions were predicted as 42 °C extraction temperature, 4.3% amount of co-solvent (ethanol) and 7.48 h extraction time with 0.338 ± 0.015 g UA/100g lichen response value. The supercritical extraction of the lichen was performed three times at the optimum conditions, and the response was determined as 0.372 ± 0.022, which was compatible with theoretical model value. The relationship between predicted and experimental values verified the accuracy of the model with points clustering around the diagonal line (Figure 5).

## 4. Conclusion

In conclusion, *U. longissima* lichen was extracted in supercritical carbon dioxide (SC-CO_2_) successfully. Box-Behnken design (BBD) of response surface methodology (RSM) was applied to obtain the high amount of UA in the lichen. The optimum conditions were determined as 42 °C extraction temperature, 4.3% amount of co-solvent (ethanol) and 7.48 h extraction time. The recommended model was acceptable, and the theoretical and experimental response values, which realized at optimum supercritical conditions were compatible.
